# Predictive value of body mass index (BMI) and determination of optimum cut-off point in the diagnosis of endometrial hyperplasia in pre-menopausal women with abnormal uterine bleeding 

**DOI:** 10.22088/cjim.15.1.9

**Published:** 2024

**Authors:** Sara Alizadeh Garna, Shahla Yazdani, Hadis Musavi, Mohammad Ranaei, Karimollah Hajian, Zinatossadat Bouzari

**Affiliations:** 1 Student Research Committee, Babol University of Medical Sciences, Babol, Iran; 2 Infertility and Reproductive Health Research Center, Health Research Institute, Babol University of Medical Sciences, Babol, Iran; 3 Clinical Research Development Unite of Rouhani Hospital, Babol University of Medical Sciences, Babol, Iran; 4 Social Determinants of Health Research Center, Health Research Institute, Babol University of Medical Sciences, Babol, Iran

**Keywords:** Abnormal uterine bleeding, Endometrial cancer, Endometrial hyperplasia, Body mass index, Infertility.

## Abstract

**Background:**

The suitable BMI cut-off point in persons with endometrial cancer or hyperplasia with abnormal uterine bleeding was investigated in this study.

**Methods:**

This case-control research was conducted on 1470 women with abnormal uterine bleeding in Ayatollah Rouhani Hospital,Babol between 2010 and 2012, with 312 participants included in the study. In terms of uterine biopsy results, patients were split into six groups: simple hyperplasia without atypia, simple hyperplasia with atypia, complicated hyperplasia with atypia, complex hyperplasia without atypia, endometrial cancer, and normal persons.

**Results:**

The mean age and BMI of patients in these three groups were not significantly different (P equal to 0.081 and 0.435, respectively). The kind of disease exhibited a strong relationship with menstruation (P 0.001). The body mass index (BMI) values ​​did not have significant levels under the curve to determine the appropriate cut-off point in the diagnosis of hyperplasia plus endometrial cancer and endometrial cancer alone (P 0.380 and 0.124, respectively) and hyperplasia alone (P = 0.920). Based on logistic regression, age 50 years and older and irregular menstruation were significant with OR equal to 2.36 and 2.09 (P = 0.011) and HTN with OR equal to 0.44 (P = 0.026), respectively.

**Conclusion:**

BMI has little predictive value in the detection of endometrial cancer or hyperplasia, according to the findings, and other diagnostic and screening modalities should be utilized instead. The findings backed up the theory that old age and irregular menstruation are linked to an increased risk of endometrial cancer.

After breast, lung, and colorectal cancers, endometrial carcinoma is the fourth most frequent cancer in women and the eighth largest cause of death from cancer. Endometrial cancer affects roughly 2% to 3% of women at some point during their lives (1). Non-ovulatory cycles raise the risk of infertility and a history of irregular menstruation (prolonged exposure to estrogen without sufficient progesterone). Endometrial cancer risk increases by 2.4 times in women who menopause beyond age 52 compared to those who menopause before age 49, which is likely due to extended uterine exposure to menstrual cycles associated with progesterone shortage (2). Those who are 21 to 50 pounds overweight have a threefold increased risk of endometrial cancer, while women who are above 50 pounds overweight have a tenfold increased risk (excess estrogen as a result of environmental conversion of androstenedione produced by aromatization of fat in the adrenal gland).

Those who are 21 to 50 pounds overweight have a threefold increased risk of endometrial cancer, while women who are above 50 pounds overweight have a tenfold increased risk (excess estrogen as a result of environmental conversion of androstenedione produced by aromatization of fat in the adrenal gland). The incidence of endometrial cancer is expected to rise in the next years as a result of the obesity pandemic in Western nations, as well as the rising frequency of insulin resistance and metabolic syndrome (2).

The most prevalent reason for a gynecologist referral is abnormal uterine bleeding (3, 4). Invasive techniques such as endometrial biopsies and hysteroscopy are frequently used. A typical pattern of bleeding in non-pregnant women, women of reproductive age after menarche that has persisted more than 6 months, is considered this condition (5). Endometrial hyperplasia with atypia is frequently associated with or precedes endometrial cancer. Anovulatory cycles, which are common in premenopausal women, polycystic ovarian syndrome, and obese women, are hypothesized to be caused by prolonged stimulation of the endometrium in the presence of estrogen alone (6). Anthropometric, dietary, physical activity, medical history (such as diabetes), hormone therapy, menstrual history, and smoking studies have all been done on endometrial cancer risk factors (7). Patients over 45 years of age and young women with a history of substantial estrogen usage should get an endometrial biopsy (8). Obesity is the most major risk factor for hyperplasia or endometrial cancer, according to several studies (9-13). Although the majority of endometrial malignancies develop after menopause, little data on the risk of obesity in hyperplasia and endometrial cancer in symptomatic young women before menopause is available (14). Obesity and endometrial biopsy in patients with abnormal premenopausal uterine bleeding have received little research, and the majority of studies have focused on the cut-off age for endometrial examination in women with abnormal premenopausal bleeding (15, 16). Overweight and obesity have grown dramatically in many industrialized and developing nations during the last two decades, affecting people of all ages, genders, racial and ethnic groupings, incomes, and educational levels (17). Studies have shown that overweight and obesity are also common in Iran (18, 19). Obesity and weight increase are prevalent in 34% and 40% of Iranian women, respectively, and are much greater in women than in males (20). In comparison to Europeans, Asians have a lower body mass index than the World Health Organization (WHO) cut-off mark for BMI, according to research (21). Obesity is linked to greater estrogen levels in postmenopausal women, which may contribute to an increased risk of endometrial cancer (22). Esmer's study was carried out to evaluate the right age for endometrial biopsy in women with abnormal uterine bleeding (16). Due to an increase in overweight and obesity in Iran, as well as a lack of studies in Iran to determine the predictive power of BMI in the diagnosis of hyperplasia or endometrial cancer in premenopausal women with abnormal bleeding, and a lack of studies in Iran to determine the predictive power of BMI in the diagnosis of hyperplasia or endometrial cancer in premenopausal women with abnormal bleeding We decided to do research in this area. The practical purpose of this study was to determine the predictive value of body mass index (BMI) and determine the optimum cut-off point in the diagnosis of endometrial hyperplasia or cancer in premenopausal women with abnormal uterine bleeding, so that a marker can be provided to facilitate the diagnosis.

## Methods

During 2010-2018, a case-control research was conducted on all women under the age of 55 who were sent to Ayatollah Rouhani Hospital in Babol for abnormal uterine bleeding and underwent endometrial biopsy. The Research Ethics Committee of Babol University of Medical Sciences granted authorization for this study, which was carried out in accordance with the ethical code (IR.MUBABOL.HRI.REC.1397.180). Patients with hyperplasia or endometrial cancer as a histological diagnosis are in the case group, whereas patients with fibroids, secretory endometrium, proliferative endometrium, and other benign instances are in the control group. Patients having a positive B-HCG test, a history of endometrial cancer, a history of hormone treatment, or insufficient data were all excluded from the study.

**Figure 1:**
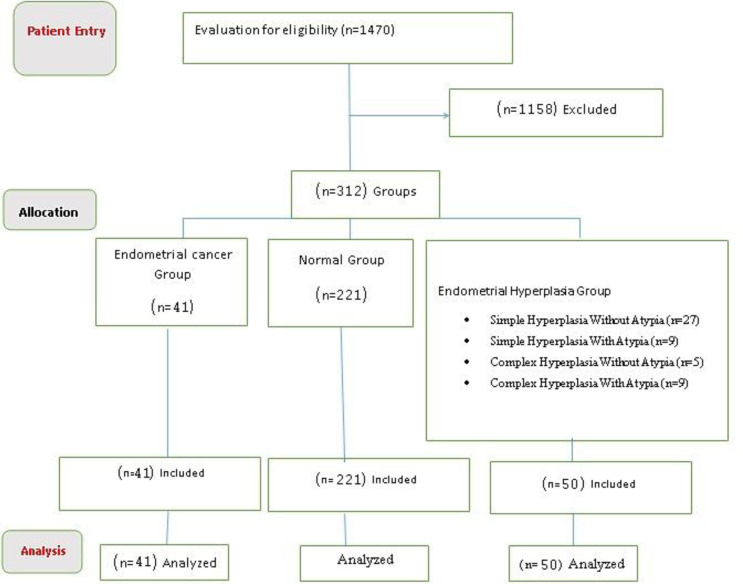
Flowchart of sampling and group assignment

Dilatation and curettage or hysteroscopy were used to collect samples under general anesthesia. To limit the mistake rate, the samples were analyzed in the pathology department of Ayatollah Rouhani Hospital, and all slides relating to the patients were checked by a pathologist. Participants' information was taken from the patients' medical records, which comprised demographic and clinical information. Age, height, weight, parity, history of menstruation, infertility (more than 12 months), medical history (history of endometrial cancer, breast, colorectal, diabetes, hypertension, history of polycystic ovaries, smoking, alcohol), and family history were among the clinical data recorded (endometrial, breast, colorectal cancer). The histology result of the hysterectomy was deemed the ultimate outcome if it was conducted 6 months following therapy. Finally, if the patient was diagnosed with endometrial hyperplasia and was treated with progesterone, the patient underwent hysterectomy, and the histological result of normal hysterectomy was reported, the final outcome of the first biopsy was evaluated (figure1). 

**Initial biopsy:** Simple hyperplasia, complex hyperplasia with atypia, complex hyperplasia without atypia, and endometrial cancer are examples of abnormal pathology. Proliferative endometrium, secretory endometrium, irregular proliferation, inflammation, endometrial polyps, and endometrial atrophy were all benign pathologies. The sensitivity and specificity of histopathology results for each group (benign pathology and aberrant pathology) were determined using the BMI and age cut-off points, respectively. Abnormal histopathology data were used to assess risk variables in both groups.

**Data validity:** A gynecological assistant evaluated and documented all patient information in this research. The pathologist in charge of the study looked over the slides and pathology results. All sample instances were also carried out by the study's lead obstetrician and gynecologist.

**Statistical Analysis:** The data were described and analyzed using SPSS software Version 26. The mean and standard deviation were used to represent quantitative variables, whereas frequency and percentage were used to express qualitative ones. To study the link between quantitative variables, the Pearson correlation coefficient was utilized. The chi-square test and, if necessary, Fisher's exact test were performed to evaluate the association between qualitative variables. The ROC curve was used to determine the link between individual and clinical factors with hyperplasia and endometrial cancer, as well as the optimal cut-off value for BMI of hyperplasia or endometrial cancer. A statistical significance threshold of less than 0.05 was used.

## Results

This study was conducted on women with abnormal uterine bleeding. Patients were split into six groups: simple hyperplasia without atypia, simple hyperplasia with atypia, complicated hyperplasia with atypia, complex hyperplasia without atypia, endometrial cancer, and normal persons.

**Analysis of demographic characteristics:**

**Assessing the age of patients:** Table 1 shows the age distribution of individuals based on histological findings. The mean and standard deviation in simple hyperplasia group without atypia equal to 46.70±5.07years, in simple hyperplasia group with atypia equal to 42.89±6.25years, in complex hyperplasia group with atypia 45.89±5.18 years, 46.20±6.14 years in the complex without atypical hyperplasia, 57.71±66.20 years in the endometrial cancer group and 44.70 ± 6.18 years in the normal group. Based on the analysis of variance test, there was no significant difference between the mean ages in different groups (P = 0.08).

 However, multiple analysis by Tukey method showed a significant difference between the normal and cancer groups (P = 0.025).

**Table 1:** Mean age (±standard deviation) and number of samples in different histological groups

**Group**	**Number**	**Standard deviation** ** ± ** **Mean**	**P-value**
**Simple Hyperplasia With Atypia**	9	42.89±6.25	**0.08**
**Complex Hyperplasia Without Atypia**	27	46.70±5.07	
**Complex Hyperplasia With Atypia**	9	45.89±5.18	
**Complex Hyperplasia Without Atypia**	5	46.20±6.14	
**Carcinoma**	41	57.71±66.20	
**Normal**	221	44.70±6.18	

* According to Tukey test, the letters a and b are significant relative to each other (P = 025).

**Figure 2:**
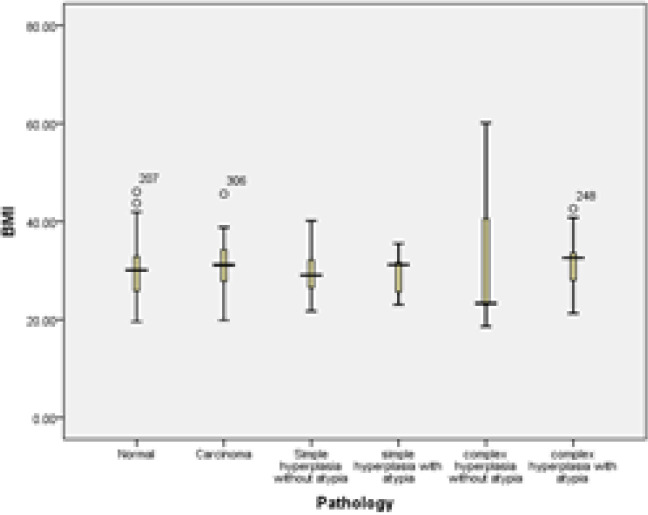
Mean distribution of BMI in different pathology groups by types of hyperplasia

**Evaluation of patients' BMI** **:** Patient groups were compared by merging hyperplasia groups with normal and cancer individuals; the mean and standard deviation were 30.21 ± 6.86 in the hyperplasia group, 31.07 ± 5.55 in the cancer group and 29.90 ± 5.03 in the normal group (figure2). There was no correlation between mean BMI in different groups (P = 0.435). Also, in multiple comparisons by Tukey method, these groups did not show a significant difference (p> 0.05). In table 2, the frequency and percentage of patients with normal BMI (less than 24.9 kg / m2), overweight (between 25.9 kg / m2) and obese (more than 30 kg / m2) in patients with The pathology of hyperplasia, cancer and normal were examined. Based on this, no difference was observed between the weight groups (P = 0.694).

**Table 2:** Frequency of BMI categories (kg / m2) based on the type of pathology of patients

**Group**	**Frequency** ** (%)**			**P-value**
	**Normal** **≤24.9**	**Overweight** **25-29.9**	**Fat** **≥30**	
**hyperplasia**	8(17.8)	13(28.9)	24(53.3)	0.69
**Cancer**	3(7.3)	15(36.6)	23(56.1)	
**Normal**	29(13.7)	70(33)	113(53.3)	

**Descriptive study of other demographic variables of patients:** Table 3 shows the demographic and clinical information by histology of patients (by integrating hyperplasia groups into a general group). As can be seen, the relationship is significant for all variables where the p-value is less than 0.05.

**Figure 3:**
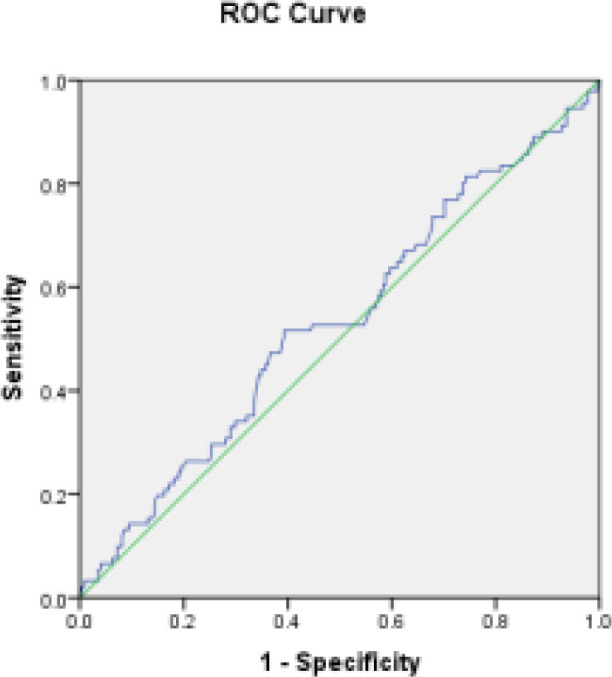
ROC curve of cancer patients and hyperplasia compared to normal individuals

**Inferential analysis of data:** BMI values in the cancer and hyperplasia groups compared to the normal group show that the area under the curve (AUC) is 0.532 and is not statistically significant (P = 0.380); the 95% confidence level of this curve was 0.60 (specificity) and 0.46 (sensitivity) (figure3).

**Table 3:** Frequency and percentage of fertility characteristics and underlying disease of patients according to different pathologies

**Variable**		**Frequency** ** (%)**			**P-value**
		Normal	Cancer	Hyperplasia	
**Gravid**	**0**	13(5.9)	7(17.1)	2(4)	0.019
	**1**	19(8.6)	5(12.2)	5(10)	
	**2**	67(30.3)	6(14.6)	7(14)	
	**3**	76(34.4)	12(29.3)	18(36)	
	**4 and more**	46(20.8)	11(26.8)	18(36)	
**Parity**	**0**	15(60.8)	8(19.5)	2(4)	0.002
	**1**	28(12.7)	6(14.6)	6(12)	
	**2**	85(38.5)	7(17.1)	15(30)	
	**3**	75(33.9)	10(24.4)	17(34)	
	**4 and more**	18(8.1)	10(24.4)	10(20)	
**Live**	**0**	16(7.2)	8(19.5)	2(4)	0.004
	**1**	28(12.7)	6(14.6)	6(12)	
	**2**	85(38.5)	8(19.5)	15(30)	
	**3**	75(33.9)	10(24.4)	17(34)	
	**4 and more**	17(7.7)	9(22)	10(20)	
**Abort**	**0**	156(70.6)	35(85.4)	31(62)	0.043
	**1**	53(24)	3(7.3)	11(22)	
	**2 and more**	12(5.4)	3(7.3)	8(16)	
**Cesarean**	**0**	129(58.4)	33(80.5)	30(60)	0.009
	**1**	45(20.4)	6(14.6)	4(8)	
	**2 and more**	47(21.2)	2(4.9)	16(32)	
**Normal vaginal delivery NVD)** **)**	**0**	69(31.2)	13(31.7)	18(36)	0.051
	**1**	28(12.7)	5(12.2)	5(10)	
	**2**	64(29)	4(9.8)	9(18)	
	**3**	47(21.3)	11(26.8)	11(22)	
	**4 and more**	13(5.8)	8(19.5)	7(14)	
**Infertility**	**Yes**	15(6.8)	7(17.5)	3(6)	0.061
	**No**	206(93.2)	33(82.5)	47(94)	
**Menstruation**	**Regular**	120(54.3)	11(26.8)	17(34)	0.001
	**Irregular**	86(38.9)	20(48.8)	31(62)	
	**Menopause**	15(6.8)	10(24.4)	2(4)	
**Diabetes**	**Yes**	24(10.9)	8(19.5)	6(12)	0.298
	**No**	197(89.1)	33(80.5)	44(88)	
**Hypertension**	**Yes**	60(27.1)	8(19.5)	11(22)	0.493
	**No**	161(72.9)	33(80.5)	39(78)	

**Figure 4:**
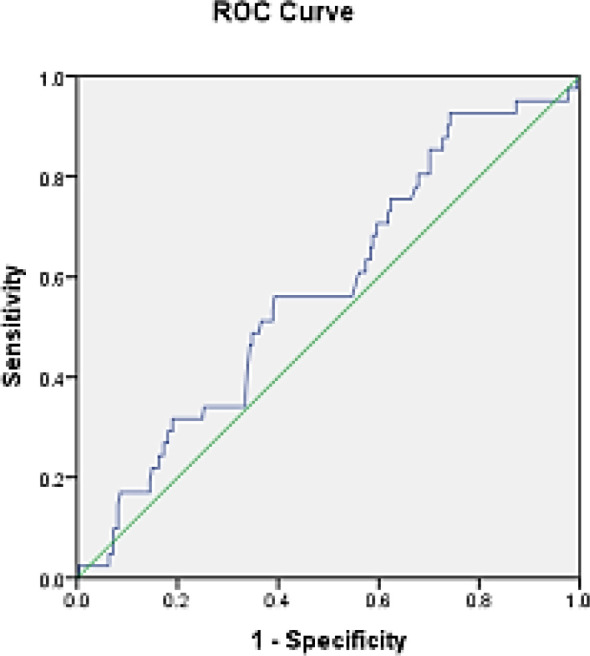
ROC curves of cancer patients compared to normal people

BMI values in the cancer group compared to the normal group show that the area under the curve (AUC) is 0.576 and is not statistically significant (P = 0.124); the 95% confidence level of this curve was 0.67 (specificity) and 0.48 (sensitivity) (figure4).

**Figure 5:**
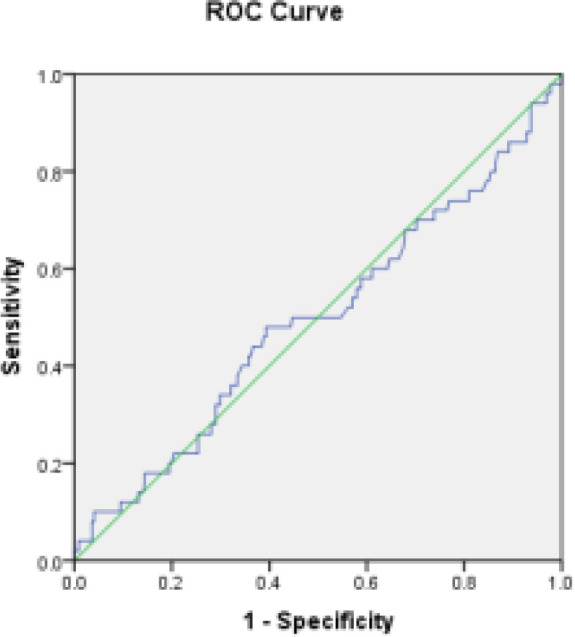
ROC curve of hyperplasia individuals compared to normal individuals

 BMI values in the hyperplasia group compared to the normal group show that the area under the curve (AUC) is 0.495 and is not statistically significant (P = 0.920); the 95% confidence level of this curve was 0.59 (specificity) and 0.40 (sensitivity) (figure5).

**Logistic regression:** In table 4, logistic regression analysis was performed to determine the odds ratio of malignancy or hyperplasia. Based on the findings, age 50 years and older and irregular menstruation with OR equal to 2.36 and 2.09, respectively, were significant (P =0.011) And HTN with OR equal to 0.44 were significant (P = 0.026). 

**Table 4:** Results of logistic regression analysis on influential variables in uterine pathology

**Variable**		**OR**	**%95CI**	**P-value**
**BMI**	Overweight vs. normal	0.99	0.42-2.33	0.978
	Obesity vs. normal	1.17	0.51-2.67	0.712
**Age**	Less than 59	1	---	---
	50 and more	2.36	1.22-4.6	0.011
**Menstruation**	Irregular vs. regular	2.09	1.19-3.68	0.011
	Menopause vs. regular	1.98	0.71-5.56	0.194
**Hypertension**	Infection versus non-infection	0.44	0.22-0.91	0.026
**Diabetes**	Infection versus non-infection	1.53	0.69-3.38	0.292

## Discussion

The In this study, the BMI of people who have abnormal uterine bleeding (AUB) was compared to that of healthy people, those with hyperplasia, and people with endometrial cancer. The groups of hyperplasia, endometrial cancer, and normal people were not substantially related with mean BMI in the initial study. In addition, when comparing different BMI categories with pathological groups, no significant link was discovered. The values below the BMI curve were not significant in comparison to cancer patients and patients with hyperplasia with normal people, cancer patients solely with normal people, and individuals with hyperplasia exclusively with normal people, respectively, in the following stage. Endometrial cancer or endometrial hyperplasia with a BMI more than 40 kg/m2 is substantially related with irregular uterine bleeding in young women and adolescents, according to Rosen's research (23). 

This study included patients aged 15 to 25 years old, as well as 71 patients and 57 healthy persons, which differed from our study in this regard. In general, the mean BMI in this research was significantly higher than ours. BMI values were shown to be unrelated to the kind of pathology of the illness in our study, both in terms of average and area under the curve, as well as categorization. However, a total of 15 patients in three pathological groups in our investigation had a BMI of 40 or above, which may have influenced the outcomes. Another study demonstrated a link between a BMI of 30 kg/m2 and endometrial cancer or hyperplasia (24). This study's target population and sample size were similar to ours. However, BMI was not identified as a predictor of endometrial cancer in our investigation, and the findings showed that BMI was not clearly connected with endometrial cancer or hyperplasia. As a result, this research contradicted our findings. 916 patients with AUB were investigated in the experiment of Wise. Patients with hyperplasia or malignancy who had a BMI of greater than 30 had a considerably higher frequency than the control group (25) 

There was a considerable variation in sample size between the wise research and our investigation. In addition, our study found no significant association between BMI and disease pathology, which contrasted with the study of Wise. Individuals aged 50 years and older, with an odds ratio of 2.36 (P = 0.011), and individuals with irregular menstruation, with an odds ratio of 2.09 (P = 0.011), were found to be significantly at risk for endometrial cancer in a study of age of patients participating in the study by logistic regression. Rosen's research focused on the link between polycystic ovary syndrome (PCOS) and endometrial cancer (26). 

It was shown that whereas smokers with a history of PCOS were not directly linked to endometrial cancer or hyperplasia, those with a BMI more than 30 kg/m2 who were smokers or had a history of PCOS had a higher risk of endometrial hyperplasia and cancer. Although there was no significant link between BMI and endometrial cancer in our investigation, those with irregular menstruation (such as PCOS) were identified as having an increased risk of endometrial cancer. A total of 1120 premenopausal individuals were investigated recently. Patients with a BMI of 30 or above were advised to get more than 30 endometrial biopsies to rule out pathological issues in this research. They discovered that BMI was a powerful predictor of endometrial cancer in women under 45, and that it also quadrupled the chance of these pathological diseases in women over 45 (27). In pathological instances of endometrial, a rise in age of more than 45 years was cited as a risk factor, which was consistent with our findings. In our study, however, the link between BMI and endometrial cancer was fully ruled out. The average age and BMI of cancer patients were substantially higher than healthy people in the Khalaf’s research. They looked at postmenopausal women with abnormal uterine bleeding (AUB) (28). 

The researchers looked at 80 patients, with uterine bleeding in postmenopausal women as the criteria for admission. In this regard, their research contradicted our findings. In addition, only old age was found to be a risk factor for endometrial cancer in our study, while BMI was not linked to endometrial cancer. Endometrial cancer or hyperplasia was not linked to monthly abnormalities or high blood pressure, according to Giannella et al.. In addition, there was no substantial link between infertility and this condition. Infertility, monthly abnormalities, and high blood pressure were all linked to an increased risk of endometrial cancer or hyperplasia, according to the findings of logistic regression (24). 

Hypertension was shown to be a protective factor against endometrial cancer in our study (odds ratio 0.44; P equal to 0.026 in risk factor analysis); additional research on this topic is needed.

 In this regard, Giannella's study differed from ours, although the two studies were comparable in terms of irregular menstruation and endometrial cancer. The biggest limitation of this investigation is given by the fact that the BMI already can effected by thyroid disorder which didn’t considered in this study. Another great limitation of being retrospective.

 The low small number of subjects with premalignant or malignant endometrial pathology due to low prevalence of EC. Moreover, AUC ROC method was used to cut-off report which could be different from the ideal threshold with involvement of all the risk factors and patient outcomes. So, a study with high samples will be more promising threshold and diagnostic model of endometrial hyperplasia in pre-menopausal women with abnormal uterine bleeding**. **In this study, each case of study has histological examination as a reference standard and also clinical variables which were measurable so there were no missing data and these factors can be the strengths of study.

BMI has little predictive value in the detection of endometrial cancer or uterine hyperplasia, according to the findings, and other diagnostic and screening modalities should be utilized instead. The findings backed up the theory that old age and irregular menstruation are linked to an increased risk of endometrial cancer. People over the age of 50, as well as those with irregular periods, were at risk for endometrial cancer, according to logistic regression research. The association between high blood pressure and the preventive effect against endometrial cancer, which is based on the findings of logistic regression analysis, is also something that needs to be looked into further.
